# Biases in small RNA deep sequencing data

**DOI:** 10.1093/nar/gkt1021

**Published:** 2013-11-05

**Authors:** Carsten A. Raabe, Thean-Hock Tang, Juergen Brosius, Timofey S. Rozhdestvensky

**Affiliations:** ^1^Institute of Experimental Pathology (ZMBE), University of Muenster, Von-Esmarch-Strasse 56, 48149 Muenster, Germany and ^2^Advanced Medical and Dental Institute (AMDI), Universiti Sains Malaysia, 13200 Penang, Malaysia

## Abstract

High-throughput RNA sequencing (RNA-seq) is considered a powerful tool for novel gene discovery and fine-tuned transcriptional profiling. The digital nature of RNA-seq is also believed to simplify meta-analysis and to reduce background noise associated with hybridization-based approaches. The development of multiplex sequencing enables efficient and economic parallel analysis of gene expression. In addition, RNA-seq is of particular value when low RNA expression or modest changes between samples are monitored. However, recent data uncovered severe bias in the sequencing of small non-protein coding RNA (small RNA-seq or sRNA-seq), such that the expression levels of some RNAs appeared to be artificially enhanced and others diminished or even undetectable. The use of different adapters and barcodes during ligation as well as complex RNA structures and modifications drastically influence cDNA synthesis efficacies and exemplify sources of bias in deep sequencing. In addition, variable specific RNA G/C-content is associated with unequal polymerase chain reaction amplification efficiencies. Given the central importance of RNA-seq to molecular biology and personalized medicine, we review recent findings that challenge small non-protein coding RNA-seq data and suggest approaches and precautions to overcome or minimize bias.

## INTRODUCTION

Based on the detection of open reading frames (ORFs), entire transcriptomes are differentiated into two main RNA classes. The first comprises all protein coding messenger RNAs (mRNAs), and various subclasses of non-protein coding RNAs constitute the second (1,2). Although non-protein coding RNAs do not encode proteins, they are key players in controlling diverse biochemical pathways. Functions carried out by non-protein coding RNAs might be exerted by the RNA itself or in complex with proteins (1–3). RNA size criteria to define small non-protein coding RNA (The size cutoff for defining small RNAs used to be about 500 nt to include small RNAs, such as SRP RNA (300 nt), 7SK RNA (332 nt), MRP RNA (287 nt) and small nuclear RNAs. Large or long RNAs were defined as being in the size range of mRNAs but devoid of apparent ORFs. The first publications on miRNAs correctly designated them as tiny RNAs to discriminate them from the larger small RNAs (4,6–8)) remain a matter of debate (4); however, in eukaryotes, size ranges of 10–500 nt are generally accepted (5). Small non-protein coding RNAs participate in the regulation of many cellular activities, including replication, chromosome modification, transcription, splicing and translation, in various organisms (6,7). Expression of many non-protein coding RNAs is subject to tight spatiotemporal control (8). Therefore, in addition to non-protein coding RNA discovery, it is of major importance to validate their corresponding expression profiles. Of late, research on non-protein coding RNAs has focused on discovery and functional analysis of micro RNAs (miRNAs), a vast class of ∼22 nt-long non-protein coding RNAs, detected in multicellular and most unicellular eukaryotes (9–12). They are involved in the fine-tuned regulation of various biochemical pathways by modulating gene expression. The majority of miRNAs exert their function via base complementarities to mRNA targets. Depending on the degree of complementarity, miRNAs complexed with proteins (RISC, RNA-induced silencing complexes) down-regulate translation or trigger RNA degradation (7,13). In addition to these posttranscriptional mechanisms of action, novel nuclear functions for miRNAs are also suggested (14,15). Furthermore, the use of miRNAs as potential biomarkers to monitor progression of disease or treatment has been reported (16,17).

The high capacity and comparably low cost of modern deep-sequencing analysis suggest that high-throughput, small non-protein coding RNA sequencing (small RNAseq or sRNA-seq) might be a superior tool when applied to personalized medicine. The commonly used microarray-based analysis has to compensate for differences in hybridization efficacies in order to ensure true representation of relative expression levels. Hybridization-based assays must also be sensitive enough to permit isoform-specific miRNA detection. Such requirements are sometimes hard to attain since individual miRNAs often display very different melting temperatures (18). Furthermore, the quantification of low-abundance miRNAs by micro-array is often complicated due to low signal to noise ratios. RNA-seq is particularly suited to investigating transcripts of low abundance (8,17,19). Deep sequencing is therefore thought to ultimately widen the dynamic range of RNA quantification (20–23), and the digital nature of RNA-seq reduces noise classically associated with hybridization-based approaches. In addition, RNA-seq does not depend on prior genome annotation and enables mapping of RNAs at single nucleotide resolution (23,24). Because probe/target-specific hybridization kinetics require expensive normalization to compare results across platforms and to rank RNA levels according to relative abundance, RNA-seq is generally considered to simplify RNA quantification (25). In general, RNA-seq is widely regarded as an appropriate tool to characterize entire transcriptomes, and it has proven to be of particular value in small non-protein coding RNA discovery (sRNA-seq methods) (8,19,24–26). Recent systematic investigations, however, revealed severe method-dependent distortions in, for example, miRNA quantification (27,28). Based on identical starting material, the analyses demonstrated that alternative methods in cDNA construction resulted in entirely different miRNA expression level profiles. Surprisingly, the choice of sequencing platform contributed little to the differences reported. Library replicates to test for reproducibility yielded comparable results, indicating that data distortion was likely caused by differences inherent to cDNA construction protocols (27). Artificial RNA test sets containing equal amounts of 473 synthetic human miRNAs were quantified by digital gene expression analysis. Throughout the study, a non-uniform distribution of the miRNAs was uncovered; the corresponding values of miRNA expression differed by up to four orders of magnitude between datasets (27). Until recently, sources of bias that might distort sRNA-seq data were not considered. Enzymes involved in RNA end-modification are obvious candidates for causing bias in relative expression levels (29); however, the subsequent steps of reverse transcription and polymerase chain reaction (PCR) amplification are also likely to display template preferences, thereby favoring the amplification of some RNAs over others. In particular, the respective RNA G/C-content greatly influences rates of cDNA synthesis and is also responsible for template-specific preferences in PCR amplification (30–32). Not surprisingly, a recent analysis demonstrated that RNA secondary structure and RNA–adapter cofolding drastically influenced RNA ligation efficacies (33).

Here, we review recent data indicating various sources of bias in sRNA-seq inherent to the common multiple experimental steps involved in library preparation. Experimental strategies and methods to overcome or minimize such distortion in expression levels are suggested wherever possible. Application of sRNA-seq-related methods in mRNA deep sequencing and personalized medicine are also discussed.

## RNA END-MODIFICATION

**Figure 1.**
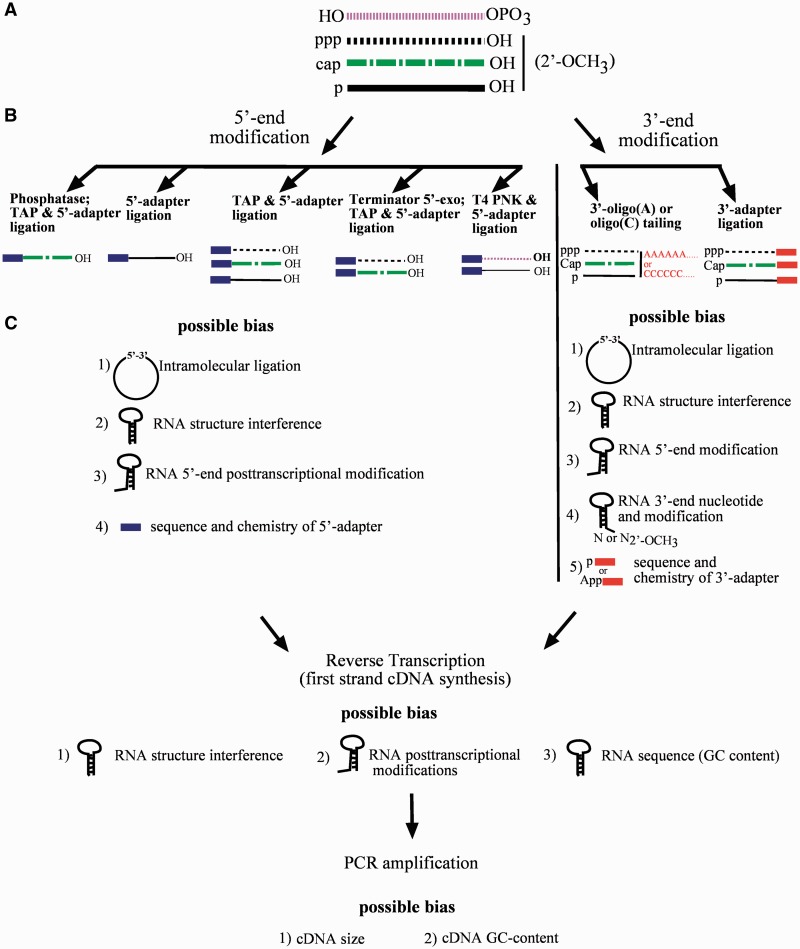
Illustration of the steps involved in cDNA construction, including potential sources of bias. (**A**) The starting pool of non-protein coding RNAs with different 5′- and 3′-end modifications schematically indicated by different line types. Abbreviations for the various modifications: OH: hydroxyl, OPO_3_: 2′-3′-cyclic phosphate, ppp: triphosphate, p: monophosphate, cap: cap, and 2′-OCH_3_: 2′-*O*-methyl. (**B**) The left panel depicts different enzymatic pre-treatments prior to RNA 5′-end ligation to enrich for different RNA subtypes. From left to right: RNA without any pretreatment (5′-adapter ligation); RNA pretreated with tobacco acid phosphatase (TAP); RNA pretreated with Terminator™ 5′-phosphate-dependent exonuclease and TAP (Terminator 5′-exo; TAP); RNA treated with T4 polynucleotide kinase (T4 PNK). RNA classes accessible for adapter ligation after the respective 5′-end pretreatments are schematically represented below each pretreatment. The right panel depicts subtypes of RNA classes accessible for 3′-end tailing (-oligo(A) or –oligo(C) tailing) and adapter ligation. (**C**) Possible biases associated with RNA 5′-(left) and 3′-(right) end-modifications and with the subsequent steps of cDNA construction.

The analysis of full-length non-protein coding RNAs in sequencing projects requires RNA end-modification or equivalent strategies to ensure identification of native RNA termini as a precondition for cDNA construction (29). Irrespective of the ensuing protocol, RNA 3′-ends are subjected to enzymatic modification to add either homopolymer stretches or synthetic adapter oligonucleotides (Figure 1; Supplementary Data and Supplementary Table S1). The template-independent addition of oligo(C) or (A) stretches is catalyzed by *Escherichia coli* poly(A) polymerase. Alternative strategies of RNA end-modification rely on RNA ligation to introduce adapter oligonucleotides. RNA ligation is also applied to modify RNA 5′-ends.

### RNA 3′-tailing

Homopolymer addition catalyzed by *E. coli* poly(A) polymerase is a well-established approach (34,35). The enzyme accepts all nucleotide triphosphates (NTPs) as substrates, and catalyzes the template-independent addition of nucleotide monophophate (NMP) to RNA 3′-termini. Alternative procedures that employ the corresponding yeast nuclear counterpart (Poly(U) polymerase, PUP) are more of a theoretical interest (35,36). Sources of the potential biases influencing RNA-3′-end tailing are discussed below.

#### Substrate preferences of E. coli Poly(A) polymerase

*In vitro*, bacterial polyadenylation is known to reduce the biological halftime of targeted RNAs (37). Sequence analyses of *in vivo*-modified RNAs strongly suggest that *E. coli* poly(A) polymerase accepts only ATP as a substrate; *in vitro* it is reported to utilize, albeit with unequal affinity, all NTPs as substrates for transfer (38). Different affinities of poly(A) polymerase toward specific NTPs influence reaction rates significantly (38). In general, UTP and GTP additions necessitate extended incubation times to reach product yields comparable to tailing with ATP and CTP. Therefore, the addition of homopolymeric (A) or (C) stretches is the most effective and common practice in cDNA generation (39–42).

#### RNA secondary structure influences RNA 3′-end tailing

*In vitro* data indicate significant reduction in the efficacy of RNA 3′-tailing caused by terminal stem loop structures (38), implying that RNA denaturation prior to RNA-tailing is of major importance to avoid bias (39). *In vitro* experiments also demonstrated that addition of three to six unpaired nucleotides might be sufficient to render the reaction secondary structure-independent (38). However, approximation of the minimal extension length needed to ameliorate RNA structure effects should not be generalized, because it is likely to be RNA structure-specific.

#### RNA primary structure influences RNA 3′-end tailing

Recently, to examine the influence of primary structure on RNA tailing efficacy, three *in vitro*-transcribed test RNAs were assayed in comparative oligo(A)- and oligo(C)-tailing reactions (39). Interestingly, after RNA denaturation, all test RNAs, irrespective of primary RNA structure, performed equally well in the C-tailing assays and proceeded up to 95% completion, as judged by shifts in mobility (calculated as percent input) (39). To examine the influences of the 3′-terminal nucleotide on RNA A-tailing, RNAs identical except for the last nucleotide were examined in oligo(A)-tailing assays, and up to 2-fold changes in the efficacy of RNA modification were reported (36). The analysis also revealed that uridine at the RNA 3′-ends was the least preferred nucleotide. However, reaction kinetics indicated that even an RNA molecule with an unfavorable 3′-terminal uridine was almost completely modified if the reactions proceeded longer (36). Therefore, increased incubation periods might compensate for substrate-specific variation in RNA 3′-tailing efficacies. Hence, the available data indicate that primary structure-related influences are less likely to bias sRNA-seq data, if the protocol relies on RNA 3′-end tailing. However, recent observations suggested that the RNA 5′-end phosphorylation state influences the resulting 3′-end homopolymer tail length of otherwise identical test RNAs (39).

#### RNA 3′-end modification impacts tailing efficiencies

The 2′-*O*-methylation of RNA 3′-ends is also reported to reduce the efficacy of RNA tailing reactions (36). The efficacy of oligo(A) addition can drop by as much as 80%, and even after extended incubation periods certain RNAs remain virtually unmodified (Figure 1). Therefore, 2′-*O*-methylation at RNA 3′-ends renders RNAs less accessible to RNA tailing (36), and such RNAs would eventually appear underrepresented in sRNA-seq. Notably, most plant miRNAs and vertebrate piRNAs are reported to be substrates for 2′-*O*-methylation (43,44). The drop in tailing efficacy and the corresponding distortion in expression level might be particularly relevant when such RNAs are subjected to RNA tailing procedures.

### RNA ligation

Today, RNA ligase-catalyzed reactions are most often utilized to modify RNA termini for cDNA construction. The genome of bacteriophage T4 harbors two different RNA ligases, T4Rnl1 and T4Rnl2. T4Rnl1 compensates for host defense mechanisms by sealing breaks introduced into the anticodon loop of host tRNA^Lys^ during infection (45,46). T4Rnl2 has been detected more recently. Although phylogenetically related to various classes of DNA and RNA ligases, the biological function of T4Rnl2 remains unknown (33,47,48). Both RNA ligases as well as derivatives of T4Rnl2 are frequently utilized in cDNA library construction. In particular, the truncated version of RNA ligase 2 has been reported to minimize side reactions (49,50). Variations in the compositions of substrates and reagents during ligation are likely to introduce biases.

#### RNA ligation-the reaction pathway

**Figure 2.**
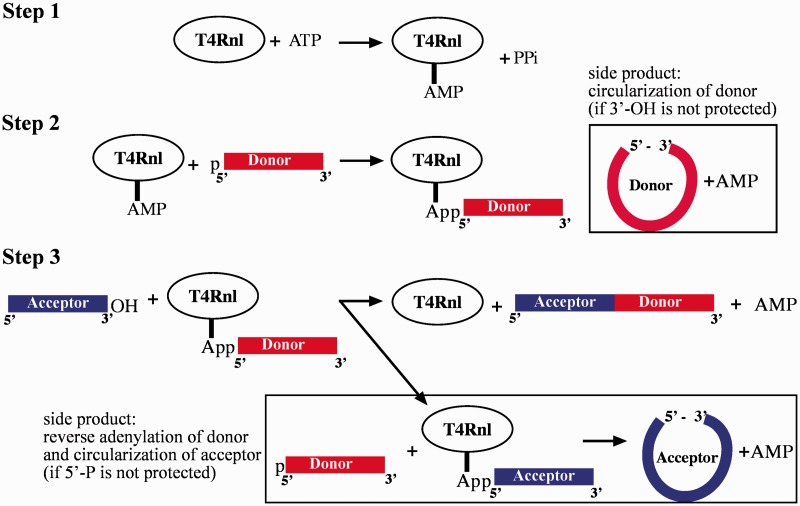
Schematic representation of the three-step mechanism of the bacteriophage T4 RNA ligase (T4Rnl) reaction with the potential side products. Encircled Arabic numbers indicate the order of ligation steps. Step 1: adenylation of the T4Rnl active site; Step 2: donor 5′-adenylation (side reaction: circularization of 3′-end-unprotected donor molecules); Step 3: phosphodiester bond formation between 3′-hydroxylated (OH) acceptor and donor molecules (side reaction: reverse adenylation of donor and circularization of acceptor molecules). PPi: pyrophosphate, p: monophosphate, AMP: adenosine monophosphate (Ap), App: 5′-adenylated termini.

RNA ligation leads to ATP-dependent 3′-5′ phosphodiester bond formation. Two molecular features are distinguished (51): donor molecules that provide 5′-monophosphorylated RNA termini (52,53) and acceptors that contain 3′-hydroxyl functional groups (52–54) (Figure 2). Two reaction intermediates are isolated, suggesting a three-step reaction mechanism. During the initial step of ligation, accumulation of monoadenylated RNA ligase is observed (55,56). The enzyme-catalyzed adenosyl-transfer to the RNA ligase requires phosphoramide bond formation and is accompanied by pyrophosphate release (Figure 2). In the second step, the AMP moiety is transferred to a 5′-monophosphorylated RNA donor molecule to form a 5′-adenylated RNA intermediate (5′-AppRNA) (52,53) (Figure 2). The 5′-5′ anhydride linkage is required to activate donor molecules for the subsequent sealing reaction. Finally, an acceptor RNA 3′-end hydroxyl group (3′-OH) attacks the alpha phosphorus of the 5′-AppRNA donor molecule, leading to covalent strand sealing and AMP release (52,53) (Figure 2). The fact that pre-adenylated donors perform efficiently in reactions conducted without ATP supplement provides formal proof of the reaction pathway and also is of major importance to practical applications (49,57). The 5′-App-modified adapters are now the reagents of choice to minimize side reactions (29,49,57).

#### Side products in ligation reactions

A side reaction common to RNA ligation is the intramolecular RNA circularization (Figures 1 and 2) (29). The reaction pathway is identical to the intermolecular ligation discussed above, and is generally preferred due to close proximity of the RNA ends. Thus, reactions that permit intra- and intermolecular products are dominated by intramolecular circularization (51,58). It is of major importance to minimize side products in RNA ligation to maintain reaction yields. Generally, the application of 5′-pre-adenylated adapter molecules in RNA 3′-end ligations is presumed to abolish RNA circularization (59,60). However, the reversal of donor adenylation as a source of circularization was recently reported (29,39,61) (Figure 2). In reactions performed with pre-adenylated donor molecules, 5′-monophosphorylated RNAs were circularized even in the absence of ATP (29,39). Remarkably, for some test RNAs, the circularization constituted the major product (39). Therefore, preferred circularization might render certain RNAs inaccessible to cDNA construction. The yield of circularization also depends on the type of RNA-ligase enzyme utilized. T4Rnl1-catalyzed reactions display the highest level of circularization (29). The tendency of T4Rnl1 to increase the amount of circular product might be explained in part by its biological function in intramolecular sealing reactions. A C-terminally truncated version of T4Rnl2 displays minimal adenyl-transfer activity while maintaining the ability to form 3′ → 5′ phosphodiester bonds (52). Ligase-mediated RNA circularization depends on AMP removal from the pre-adenylated 5′-App-adapter and its subsequent transfer to 5′-monophosphorylated RNAs; therefore, the application of truncated T4Rnl2 (T4Rnl2tr) to minimize this side reaction is highly suggested (49,50). In addition, the exchange of lysine with glutamine (K227Q) in T4Rnl2tr further reduced adenyl-transfer competence but retained activity to catalyze phosphodiester bond formation (48). Therefore, the application of T4Rnl2 derivatives is beneficial for decreasing bias in sRNA-seq analysis (29).

Alternatively, phosphatase pretreatment to generate RNA 5′-OH termini that are inert to ligase-catalyzed adenylation helps to minimize side reactions. The procedure ultimately requires the application of polynucleotide kinase to generate monophosphorylated RNA ends. However, tRNA, rRNA and mRNA processing intermediates are reported to contain 5′-hydroxylated termini (29,62). Polynucleotide kinase treatment as a necessary prerequisite to RNA 5′-adapter ligation leads to the inclusion of abundant processing intermediates in cDNA libraries. The alternative techniques to avoid RNA circularization might distort cDNA representation and are therefore likely to reduce the complexity of sRNA-seq experiments (29,62).

#### 3′-end ligation depends on RNA–adapter combination. 

RNA 3′-end ligation using T4Rnl1 was recently reported as a potential source of bias in deep sRNA sequencing (39). Comparisons of the product yields of ligation reactions were made using three test RNAs and various adapter sequences. Depending on the specific RNA–adapter combination, the products differed remarkably. Similarly, Jayaprakash *et al.* (63) reported variations in product yields depending on specific RNA–adapter combinations in Rnl2tr-catalyzed reactions. Because the RNA 3′-end ligation experiments were conducted with 5′ pre-adenylated adapters, the results also indicate that adenyl-transfer is less likely the cause of the observed bias (63). RNA 3′-adapter ligation was enhanced when adapter pools displaying nucleotide variation at the two 5′ terminal positions were used (63). Therefore, increased variability at adapter 5′-ends enhances RNA ligation yield and, in general, helps to minimize distortion in sRNA-seq (33,39,63). RNA 5′-phosphorylation also influences the efficacy of adapter ligation to RNA 3′-ends (39). The ligation efficacies of T4 Rnl1-mediated reactions were compared for test RNAs featuring different phosphorylation states at RNA 5′-ends and different adapters, including those with 5′-App-modification. Unexpectedly, RNAs that performed well when monophosphorylated, displayed weak acceptor characteristics when triphosphorylated. Therefore, the efficacy of RNA 3′-end modification might also be a function of the RNA 5′-phosphorylation state (Figure 1).

#### Ligation of adapters to modified RNAs

In ligation assays, T4Rnl1 and T4Rnl2tr performed almost identically using 3′-unmodified test RNA substrates. However, the efficacy of T4Rnl1-catalyzed reactions dropped remarkably when 2′-*O*-methylated RNAs were used (36). In contrast, T4Rnl2tr performed almost equally well irrespective of RNA modification (36). The additions of 25% (w/v) PEG 8000 to the T4Rnl2tr-mediated reactions significantly increased reaction yields (50,64). Hence, additives that increase the effective molecular concentration have a major influence on product yields, and meta-analyses of data generated by different ligases or even in different buffer compositions are error-prone.

As an aside, specific enrichment of 3′-end 2′-*O*-methylated RNAs, like the piwi-interacting RNAs (piRNAs) or plant miRNAs, is achieved by periodate treatment. The reaction relies on vicinal hydroxyl groups to oxidize sugar moieties and is accompanied by pentose ring opening. The resulting vicinal dialdehyde reacts with water and forms a chemical equilibrium with the corresponding dioxane derivative upon ring closure. The 2′-*O*-methyl group-containing RNAs are insensitive to oxidation and remain accessible for adapter ligation or tailing (65–67).

#### Impact of RNA secondary structure and adapter-RNA cofolding on ligation

Recent analysis indicates that RNA secondary structure impacts sRNA-seq bias (29). During kinetic profiling of RNA–adapter ligation, test miRNAs were trapped in non-reactive states. Denaturing the remaining non-ligated RNAs resulted in increased ligation yield, indirectly suggesting the influence of RNA secondary structure (29). To further analyze the impact of secondary structure on RNA 3′-end ligation, a pool of substrate RNAs with 21 randomized nucleotides at their 3′-ends was ligated with individual, pre-adenylated DNA adapters, and reaction yields were subsequently quantified by deep sequencing (33). To avoid bias caused by template-specific RNA 5′-end modification(s), all test RNAs contained identical 5′-termini (33). RNAs that harbored stems at their 3′-ends were underrepresented in the sequencing data, whereas RNAs containing a minimum of three unstructured nucleotides at the 3′-ends were overrepresented. The data indicate that RNA ligase-mediated reactions in general are more efficacious with RNA substrates that harbor structurally accessible 3′-ends (33).

In addition, the cofolding of RNA and adapter molecules impacts ligation (33). Adapter sequence variation that interfered with the RNA 3′-stem formation and permitted intermolecular base-pairing increased ligation yields (33). Interestingly, this data contradicts prior analysis emphasizing a predominant primary sequence-related influence on data distortion (33,63). Although the actual source of discrepancy remains to be analyzed, protocol-specific variation, probably due to changes in denaturation, is likely to have caused the observed difference (see supplementary information). Irrespective of the actual reason, the advice for minimizing this bias is identical and relies on the application of adapter pools to increase the likelihood of productive interactions (33). Thus, in addition to the RNA nucleotide composition, RNA–adapter cofolding and secondary structure-related influences contribute to sRNA-seq bias.

#### RNA 5′-end ligation*.*

The chemical properties of RNA 5′-ends display structural variations. Depending on the starting material and experimental design, it may be necessary to alter the chemistry of RNA 5′-termini prior to cDNA generation. For example, neither 5′-triphosphorylated RNAs in prokaryotes nor primary eukaryotic RNA polymerase III transcripts are accessible for direct 5-adapter ligation. Similarly, RNA polymerase II-specific trimethylguanosine cap structures render RNA 5′-ends inaccessible. Thus, enzymatic pretreatment is usually conducted to avoid bias in the starting material (Figure 1; Supplementary Data and Supplementary Table S1).

#### Specific biochemistry of RNA 5′-termini and enrichment of various RNA classes

Although the various chemical properties of RNA 5′-ends establish limitations for unbiased representation, they also permit specific enrichments (41) (Figure 1; Supplementary Data and Supplementary Table S1). RNAs that contain monophosphorylated 5′-ends and 3′-hydroxyl groups are directly accessible to cDNA generation procedures. Consequently, unaltered RNA starting material leads to its specific enrichment (Figure 1; Supplementary Table S1). In contrast, triphosphorylated or 5′-capped RNAs require TAP treatment to generate 5′-monophosphorylated RNAs and to permit 5′-end ligation.

Biochemical alternatives to specifically cleave pyrophosphate bonds in 5′-triphophorylated RNAs were reported recently (68), whereby TAP-mediated hydrolysis is substituted by polyphosphatase (69) or pyrophosphohydrolase (70,71) treatment. *In vitro* analysis to investigate the substrate specificity of *E. coli* rppH pyrophosphohydrolase indicated that pyrophosphate hydrolysis proceeds with identical efficacy, irrespective of the RNA 5′-terminal nucleotide (71). Furthermore, a subtle decline in the enzymatic activity in response to potentially inhibitory RNA 5′-terminal stem loop structures was reported (71). These strategies are particularly appealing when bacterial primary transcripts or nascent eukaryotic RNA polymerase III transcripts are central to the investigation. In addition to targeting triphophorylated RNA 5′-ends, polyphosphatase also accepts diphophorylated RNA 5′-termini to catalyze pyrophosphate bond cleavage. It should be emphasized that 5′-capped RNA polymerase II transcripts are not amenable to polyphosphatase or pyrophosphohydrolase treatment and therefore escape identification.

The application of a Terminator™ 5′-phosphate-dependent exonuclease is frequently utilized to enrich for 5′-capped and 5′-triphospharylated RNAs (41,72,73). The inherent 5′ → 3′ exonuclease activity digests 5′-monophosphorylated RNAs and leaves other transcripts unaltered (Figure 1; Supplementary Data and Supplementary Table S1). Therefore, different chemistries of RNA 5′-ends not only distort sRNA-seq, but also allow for specific enrichment protocols. Subsequent analysis permits the distinction among RNA subsets, such as primary transcripts, capped RNAs and other products of RNA 5′-end processing (41,72,73).

#### Nucleotide preference of RNA 5′ ligation

Apart from specific differences in RNA 5′-ends, the influence of the adapter sequence itself on ligation efficacy is well documented. Biochemical data indicated that ligation reactions proceeded most effectively when an adenosine residue occupied the 3′-terminal position of adapter (acceptor) molecules (51,74). Recently, adapter pools displaying sequence identity at all except the two 3′-terminal nucleotides were used to investigate sequence-dependent variation in acceptor function. Based on deep sequencing as the readout, the relative ranking of miRNA frequencies varied as a consequence of the specific adapter–RNA combinations (63). Once more, miRNA expression profiles are not only a function of true RNA abundance but also reflect varying ligation efficacies (63). Interestingly, the use of adapter pools highly improved the correlation of miRNA levels between replicates (63).

#### Influence of RNA secondary structure on RNA 5′-end ligation

To investigate potential influences on 5′-end modification derived from RNA secondary structure, ligation yields were compared among test sets of fully randomized RNAs. A remarkable underrepresentation of double-strand folds at 5′-ligation sites indicated strong preferences for single-stranded RNA substrates in ligation reactions conducted with T4Rnl1 (75).

#### Sugar moiety-dependent change in ligation efficacy

DNA-based adapter oligonucleotides perform well in RNA 3′-end ligation reactions (especially when 5′-adenylated), whereas RNA-based adapters are preferred for RNA 5′-end modification (76). Kinetic analysis of DNA-based adapter molecules for RNA 5′-ligation demonstrated slower reactions compared with their RNA counterparts (77). Recent analyses revealed more complex substrate-specific effects between test RNAs and different adapters. When DNA- and RNA-based adapters of identical sequences were investigated in ligation assays, no general preference with regard to the sugar moiety of the adapter molecule was observed in RNA 5′-end ligation (39,63). In conclusion, to avoid bias, adapter pools are recommended to display sequence variation. The chance of productive ligation might be enhanced using RNA, DNA or mixed adapter oligonucleotides (39,63).

#### Adapter barcoding influences ligation

In addition to nucleotide preferences of the reacting residues, upstream sequences also exert influence on ligation yields. Adapters harboring different Roche MID tags placed 19 nucleotides upstream from the reacting 3′-ends performed entirely differently when assayed with identical test RNAs (39). Depending on the combination of *in vitro* transcribed non-protein coding RNAs and MID-modified adapters, ligation product levels (calculated as non-protein coding RNA input) ranged from 60–70% to undetectable (39). Recent reports emphasized that barcoding for multiplex sequencing leads to significant bias in miRNA expression profiles (78–80). This bias was diminished when barcodes were introduced by PCR amplification of cDNA (78–81).

#### Quantitative considerations and barcoding

In general, the discovery of novel RNAs and analyses of transcripts that are expressed at very low levels require substantially more cDNA reads than experiments directed toward investigating differential gene expressions. Hence, the design of sRNA-seq experiments needs to take into account the rationale of the investigation. Contigs formed by continuously overlapping reads during genomic mapping procedures represent the bio-computational equivalent of individual RNA entities. Any selected read during cDNA mapping either contributes to pre-existing, already populated RNAs or might generate novel contigs. The probability of either process is a function of RNA size as well as the corresponding RNA expression value. Of course, contigs representing longer rather than shorter RNAs are more likely to be populated by chance. RNAs of higher relative expression levels are generally more densely packed with cDNA reads than RNAs that display medium or low transcription levels. Consequently, if multiplexing is chosen for economic reasons, the necessary sequencing depth must be approximated prior to sequence runs in order to generate adequate sample sizes. The two fixed parameters for estimating admissible multiplexing are therefore (i) experimental goal and (ii) lane capacity. According to Illumina® TruSeq Small RNA Sample Prep Kit guidance, 5 million sRNA-seq reads are considered appropriate for novel miRNA discovery within human-sized genomes. For miRNA profiling usually about 2.5 million reads are recommended. These approximations permit recalculation geared toward specific needs and requirements.

#### Alternative cloning strategies to avoid 5′-end modification

Because the 3′-terminal position of cDNA corresponds to the RNA 5′-ends, Pak *et al.* (82) used a technique to avoid modification of RNA 5′-termini and therefore to minimize the need for enzymatic pretreatment. The strategy introduces adapter sequences, not to RNA 5′-termini, but rather to 3′-ends of the corresponding cDNAs (82). The advantage of omitting enzymatic treatment of RNA 5′-ends might be offset by incomplete reverse transcription that precludes full-length cDNA cloning or appropriate assessment of RNA 5′-ends.

Recently, ligation to cDNA 3′-ends was investigated in more detail (36). Perfect double-stranded DNA-RNA test hybrids and single-stranded DNAs were utilized to mimic potential products of reverse transcription. All T4 DNA- and RNA-ligases, including T4Rnl2 truncated derivatives, were compared to delineate optimal conditions for cDNA 3′-end modification. Surprisingly, ligation reactions carried out with pre-adenylated DNA donor molecules strongly indicated that double-stranded DNA-RNA chimera constituted the most reactive substrates for T4Rnl1-mediated ligation. In addition, supplements of PEG 8000 strikingly influenced ligation; final concentrations of 25% PEG 8000 increased reaction yields. Identical single-stranded test cDNAs exhibited only poor substrate characteristics. The A-form-like conformation of the DNA-RNA hybrid might have compensated for the primary structure-related influences and permitted more similar ligation outcomes among substrates (36). However, the analysis examined only one test substrate and therefore might not be amenable to drawing general conclusions (36). A further interesting observation indicates that cDNA 5′-terminal nucleotides influence cDNA 3′-end ligation yields (39). As the cDNA 5′-terminal sequence is a consequence of specific RNA 3′-end modification, differences between protocols exert different influences on cDNA 3′-end adapter ligation and, in turn, might also cause expression profile bias (39).

The ‘Smart™ Approach’ (83) offers another interesting alternative to capture mature RNA 5′-ends and to avoid RNA 5′-end adapter ligation. The technology is based on the template switching capacity of Moloney murine leukemia virus (M-MLV) reverse transcriptase. Once first strand synthesis approaches the RNA 5′-end, the terminal transferase activity of M-MLV reverse transcriptase adds oligo(C)-stretches to the growing cDNA. Oligonucleotides that 3′-terminate with oligo(G) stretches serve to extend templates and permit reverse transcription to proceed. Thus, M-MLV reverse transcriptase enables the generation of cDNAs with known terminal sequence elements (83), and thereby eliminates the need to modify the RNA 5′-ends. Recently, the technology was adapted to the specific needs of Ilumina RNA-seq analysis (84).

#### Adapter–adapter ligation disturbs sRNA-seq

A side effect common to sRNA-seq protocols that rely on ligase-mediated sequential RNA end-modification, is PCR amplification of empty adapter–adapter ligation products (81,85). Adapter–dimer formation is a direct consequence of the molecular adapter excess employed to ensure sufficient product yield and might dominate in reactions where the concentration of total RNA starting material is low (81,86). Adapter molecules that are 5′-pre-adenylated remain in reaction mixtures after RNA 3′-end modification and react with RNA 5′-adapter oligonucleotides, which subsequently favors dimer formation. Therefore, purifying products away from excess adapters via gel electrophoresis is recommended (78). However, for preparation of tiny RNAs, such as miRNAs, separation of the small size difference between ligated product and adapters is not trivial. Alternative approaches, to avoid size fractionation, rely on application of complementary ‘dimer-eliminator-LNA-oligonucleotides’ (85,86). The interfering oligonucleotide is designed to overlap the adapter–adapter ligation junction and thereby distinguish between targeted side product and adapter ligated RNA (86). The complementarity of the interfering oligonucleotides extends into the RNA 3′-adapter sequence, thus blocking primer annealing during reverse transcription and, as a result, excludes adapter dimers from RNA sequencing (86). Vigneault *et al.* reported a similar strategy to overcome unintended dimer amplification (81). Pre-annealing of the RT primer after RNA 3′-adapter ligation but prior to RNA 5′-end modification efficiently decreased adapter dimer formation. The excess of non-ligated, free RNA 3′-adapters was captured by complementary reverse transcriptase primers and sequestered in less-reactive double-stranded structures (81). The latter approach permits effective reduction of dimer amplification and is reported to increase cDNA library complexity (81). Obviously, RNA tailing strategies (see above) are superior because they completely circumvent the problem of adapter-dimer formation. To avoid adapter tailing as an analogous side reaction to dimer formation, it is advisable to carry out 3′-tailing prior to 5′-adapter ligation.

Depending on the experimental goal, sRNA-seq requires varying amounts of cDNA reads (see above). Therefore, techniques to reduce the amount of reads that represent empty adapter dimers are of central importance, as they permit an increase in sequencing depth, reducing costs and ultimately enabling novel small non-protein coding RNA gene discovery.

## PCR AMPLIFICATION BIAS

Apart from novel third-generation sequencing technologies that permit direct sequence analysis of unamplified cDNA, all commonly used protocols implement PCR amplification (87). In addition, the introduction of barcode identifiers by PCR amplification may also avoid bias introduced by differences in RNA–adapter-specific ligation efficacies (78). Therefore, multiplexing of different samples during PCR is widely regarded to permit meta-analysis (78). However, even PCR amplification itself is known to introduce expression bias into datasets (30,32,88). In particular, when many different templates are amplified in parallel, variations not only in template length but also in base composition might lead to preferred template-specific amplifications (30). Extreme examples of allelic dropout due to single base-exchange were reported for PCR-mediated genotyping (89). The RNA G/C-content is especially responsible for data distortion. Generally, cDNAs of high G/C-content are less readily amplified and thus remain underrepresented. For example, read distributions calculated over the entire 28S rRNA were inversely correlated with the corresponding regional G/C-content (32). As argued earlier, base compositions of different small non-protein coding RNAs (miRNAs, piRNAs, snoRNAs, scaRNAs, etc.) vary widely. The parallel amplification of complex template mixtures that vary in their respective G/C-content is therefore a challenging procedure and likely to influence the complexity of the resulting cDNA library (30). Changes in complexity do not only influence distortion of quantitative measurements, but certain RNAs might also ‘drop out’ completely and remain unidentified. Extended periods of denaturation are considered to effectively decrease the likelihood of uneven amplification (90). PCR amplification does not only change the overall G/C-content of the resulting cDNA pool but is also reported to introduce length bias (30), which might be an important factor when RNAs of different sizes are subjected to parallel cDNA preparations. Shorter sequences of significantly lower G/C-content are likely to be overrepresented.

Recent analysis also emphasized the impact of PCR buffer compositions on PCR amplification bias (30). Remarkably, PCR-mediated bias was already observed in some cases within 15 rounds of PCR amplification (30). While the popular Phusion PCR system surprisingly performed the worst (30), data indicate that some optimized PCR buffers are capable of significantly reducing PCR-mediated distortion (30,90).

## sRNA-SEQ AND PERSONALIZED MEDICINE

In recent years, increasing attention has focused on the potential of miRNA transcriptional profiling to diagnose disease or to monitor progression and success in treatment (16,17,91). The decreasing costs of high-throughput sequence analysis favors sRNA-seq as a common tool for medical diagnostics and personalized medicine. After cell death, the total RNA of circulating cancer cells is released into the blood stream (92). These extracellular RNAs (exRNAs) are packaged within vesicles such as exosomes, where they are complexed with different proteins, for example miRNAs in the RISC complex. Vesicles protect exRNA from ribonucleases and prevent degradation. Among the various exRNAs, miRNAs are some of the most stable molecules (93). Easy accessibility of patient blood samples and the stability of miRNAs make them effective biomarkers (94). Diagnostics based on miRNA were successfully conducted on biological samples of low quality, including dried blood (95). In addition, miRNAs remain stable even when samples are subjected to repeated rounds of tissue freezing and thawing, which usually degrades RNAs (96). A further appealing feature of miRNAs in medical research is that changes in their expression profiles are observed during disease onset (97). In addition, miRNAs often display tissue-specific expression. Therefore, it might be possible to identify the cellular origin of most metastases (91), possibly providing decision guidance for appropriate treatment. However, as elaborated in detail, miRNA expression profiles detected by deep sequencing do not only reflect changes in relative expression but are also influenced by miRNA-adapter-specific ligation efficacies, especially in cost-effective parallel analyses that often require multiplexing. Different barcode introduced by ligation generate bias in miRNA representation and abundance (78). Even when incorporated by PCR, barcoding might still lead to data distortion (30–32). Third-generation sequencing techniques, such as the Helicos (In November 2012, Helicos filed bankruptcy (http://biz.yahoo.com/e/121115/hlcs8-k.html)) system, avoid PCR amplification steps in cDNA preparation and permit direct sequencing of unamplified samples (87). The RNA 3′-end modification to prime cDNA synthesis (or direct sequencing) in addition to relatively high error frequencies (∼15%) might still lead to bias and so would not be especially applicable in personalized medicine (98). Collectively, deep sequencing to monitor change in medical samples is of importance and certainly of interest, but requires resolution of serious standardization issues prior to general application.

### qRT-PCR a valid alternative to deep sequencing

Alternative techniques to quantify specific miRNA test sets rely on quantitative real-time reverse transcription PCR (qRT-PCR) (99). In particular, diagnostic screens in cancer medicine rely on the application of qRT-PCR (99–101). In cases where biomarker disease correlation is well established and when quantification of limited numbers of biomarkers is sufficient for diagnosis and to monitor disease progression or treatment, qRT-PCR is a valuable tool (99). Recently, the convenience of qRT-PCR-based medical diagnosis was suggested to make it superior in routine clinical applications (99). Interestingly, novel developments in qRT-PCR allow sample processing in 384-well formats and liken this method to cost-effective high-throughput applications (99,102). A further advantage of qRT-PCR is the reduction in downstream bio-computation (99,103). The time between qRT-PCR and diagnostic result is shorter than for sRNA-seq or microarray-based inference. However, the analysis of miRNA expression profiles by qRT-PCR is technically challenging, due to RNA-inherent pitfalls (103,104).

The miRNAs lack structural features such as mRNA poly(A) tails that could serve as class-specific enrichment or priming of reverse transcription (103). Alternative techniques for miRNA sample enrichment would rely on immune-precipitation but most of them are not suitable for typical small size samples in medical analysis (105). As mentioned above, the heterogeneity of the RNA G/C-content translates into a wide range of primer annealing temperatures for a population of miRNAs and might therefore complicate parallel analysis of multiple RNAs (103). Furthermore, the qRT-PCR template is present not only within the mature miRNA molecule but also is part of various precursor transcripts (103). It is therefore important to ensure that primary or precursor-miRNAs (pri- or pre-miRNAs) do not provide templates in qRT-PCR amplification and, as a consequence, do not contribute to the signal intensities detected. Difficulties that relate to the similarity displayed between different miRNA isoforms are mentioned above within the context of array-based inference to quantify differential gene expression.

Thus far, two alternative approaches are commonly utilized to reverse transcribe miRNA templates (103,106). miRNA-specific stem-loop primers are often used when only limited numbers of RNAs are subject to quantification. A major advantage of using miRNA-specific primers for reverse transcription is an increase in sensitivity and specificity. However, RNA 3′-end-tailing-based approaches enable bulk reverse transcription of multiple targets in parallel and are therefore advantageous when many miRNAs are analyzed (see above). A recent comparative analysis to investigate reverse transcription efficacies of the two methods uncovered only minor differences (106). The analysis involved only a limited miRNA test set, and therefore the results cannot be generalized. As an aside, the oligo d(T)-based design might also enable reverse transcription of miRNA and the corresponding mRNA target in single test tube reactions (106). Ligation-mediated PCR or padlock probes in combination with rolling circle amplification are technical alternatives for avoiding reverse transcription (107,108). The methods rely on ligation-mediated template generation and are therefore not devoid of bias (see above). Both approaches permit generation of miRNA signatures across samples and do not require elaborate analytical post-processing or expensive technical equipment (107,108). However, as elaborated here and above, reverse transcription is not devoid of template-specific bias. In particular, RNA secondary structure and RNA modifications both influence reverse transcription efficacy (see above). Similar to PCRs, reverse transcription is negatively affected by increasing RNA G/C-content.

Because the relative RNA G/C-content dictates primer Tm, qRT-PCRs are also strongly influenced by forward primer design, thereby affecting both qRT-PCR specificity and sensitivity. Altering the length of primer-template complementarity permits adjustment of the actual primer Tm. Conversely, in cases where RNA nucleotide composition would dictate very low melting temperatures, LNA-modified nucleotides might be incorporated into the primer sequence to increase the corresponding primer Tm (18). However, the design of LNA-containing oligonucleotides is not always trivial (109). For the actual measurement of miRNA in real time, two alternative fluorescent reporter molecules are frequently utilized: SYBR® Green and the Taqman® assay (103). Detection of the fluorescence signal emitted by SYBR® Green increases about 100-fold as the molecule intercalates in double-stranded DNA. However, the mechanism of DNA intercalation suggests the dye is not target-specific and therefore cannot discriminate primer dimers, unspecific products and target amplification (103). Because the intercalating dye is simply added to the assay, it is often possible to easily adapt the qRT-PCR assay to already established and validated analytical PCRs (104). In comparison, the Taqman® assay provides increased specificity, as the detection is based on sequence-specific probe–target interaction. Certainly, the qRT-PCR assays offer many advantages but require evaluation of various factors that influence and potentially bias analysis. Therefore, international standardization to ensure comparable experimental design and to permit meta-analysis among datasets is of prime importance for establishing clinical qRT-PCR-based personal medicine. The Minimum Information for Publication of Quantitative Real-Time PCR experiments guidelines established standards for better experimental practice and suggest routes to data analysis that increase reliability and data comparison (110).

## mRNA-SEQ

Deep sequencing is not limited to expression profiling of miRNA or other small non-protein coding RNAs. Even entire transcriptomes, including all protein coding and long non-protein coding RNAs, are subjected to high-throughput sequencing methods. Currently, as the length of single sequence runs is limited (from 100 to 500 nt, depending on the sequencing platform), longer RNAs require fragmentation prior to cDNA synthesis (25). Millions of small reads resulting from mRNA-seq are analyzed to identify novel transcripts or splice variants (111,112). Relative quantification of reads enables one to monitor differential gene expression among samples and to identify changes in isoform distribution (111). As with sRNA-seq, the digital nature of RNA deep sequencing is thought to simplify meta-analysis of mRNA or long non-protein coding RNA expression data (25). Some protocols for modification of RNA fragment ends rely on chemistries identical to sRNA-seq (84,113). Therefore, expression biases observed in sRNA-seq analysis are also likely to cause distortion in whole transcriptome profiling. Variations in nucleotide composition and changes in RNA secondary structure of different RNA fragments might correlate with uneven read distribution. Unequal read coverage generates uncertainty in isoform detection, and, in extreme cases, exons representing unfavorable nucleotide compositions might escape analysis. As fragmentation is suggested to generate many different, but at least partially overlapping reads, bias in mRNA-seq might be less prominent. Recently, a novel technique based on circligase was introduced to avoid RNA 5′-end ligation (114). Reverse transcription is conducted with anchored oligo(dT) primers that contain an additional sequence to permit later PCR amplification. Following first strand cDNA synthesis, circligase-mediated ligation is carried out. The resulting circular molecules are subjected to PCR amplification. The strategy is of general interest and might be a valuable tool for sRNA-seq as well.

## CONCLUSION

Bias in general describes systematic errors that reflect method-related distortion from the truth. Bias might be detected easily when data generated by different methods are subjected to analytical comparison. Because it is systematic in nature, bias is not subject to variation in repeated experiments. Here, based on biochemical and deep sequencing data analyses, we have presented various steps in sRNA-seq protocols likely to cause severe distortions in the relative expression levels of individual sRNAs.

The efficacy of RNA end-modification to permit cDNA construction depends not only on differences in strategies and reagents but also on changes in, among others, buffer compositions and additives that could increase molecular crowding. Therefore, meta-analysis of data generated with non-identical methods, reagents or even buffers is likely to be error-prone (36,39,63,78). To achieve comparable results and to evaluate relative changes in the quantification of the resulting data, experiments and analyses should be conducted under strictly identical conditions. It has to be stressed that bias in RNA-specific variation is not only restricted to inter-sample comparison, but also effects the relative ranking of intra-sample RNA expression.

In addition to the more obvious quantitative aspects of bias, there are also qualitative aspects because RNA-seq methods are also employed for RNA discovery. Individual RNAs might ‘drop out’ from detection because they are not amenable to cDNA construction under chosen conditions. Certainly, to increase the likelihood of productive RNA end-modification and increase the complexity of sRNA-seq library content, the parallel application of different strategies and reagents is required (33,39,63,79). In particular, increased variability of adapter sequences (i.e. adapter pools) helps to increase the diversity of RNAs accessed (63). A recent report emphasized the need for parallel application of different RNA- and DNA-based adapter oligonucleotides (39).

The source of expression level distortion and the failure to detect certain RNA species at all, appear not to be limited to reactions of RNA/cDNA-end modification, but also apply to cDNA amplification by PCR (30–32). Differences in reaction buffers, RNA G/C-content, secondary structures, RNA length and primers might each lead to bias during PCR. Therefore, the analysis of sRNA-seq data to examine relative changes in gene expression or identification reflects both true RNA abundance and biases related to the methods applied.

The sRNA-seq approach is also attractive to medical research. Deep sequencing might permit the screening of multiple patient samples to monitor the progression of disease or treatment. Therefore, it is urgently necessary to establish rigid international standardizations to minimize distortion and to avoid misleading conclusions during RNA-seq data interpretation. In summary, qualitative and quantitative RNomics remain more challenging than anticipated.

## SUPPLEMENTARY DATA

Supplementary Data are available at NAR Online.

## FUNDING

National Genome Research Network [NGFNIII 01GS0808 to J.B. and T.S.R.]; the Deutsche Forschungsgemeinschaft [BR 754/4-1 to J.B.]; USM Research University Grant [1001/CIPPT/81305 to T.H.T.]. Funding for open access charge: The licence was funded by Dr. Brosius’s research budget.

*Conflict of interest statement*. None declared.

## Supplementary Material

Supplementary Data
