# Supplementary material for: Biases in small RNA deep sequencing data
Source: Nucleic Acids Res. 2013 Nov 5;42(3):1414–26. doi: 10.1093/nar/gkt1021 (PMC3919602; doi:10.1093/nar/gkt1021)
Supplement: Supplementary Data [file supp_42_3_1414__index.html]

Biases in small RNA deep sequencing data — Biases in small RNA deep sequencing data — Supplementary Data 

# Biases in small RNA deep sequencing data

## Supplementary Data

files

**Files in this Data Supplement:**

- Supplementary Data - pdf file
- Supplementary Data - doc file
