# Supplementary material for: Biases in small RNA deep sequencing data
Source: Nucleic Acids Res. 2013 Nov 5;42(3):1414–26. doi: 10.1093/nar/gkt1021 (PMC3919602; doi:10.1093/nar/gkt1021)
Supplement: Supplementary Data [file supp_gkt1021_nar-01932-survey-d-2013-File005.pdf]

Supplementary Table 1. Summary of the cloning procedures used to enrich sRNA-seq libraries with different subsets of RNA species

STARTING MATERIAL	ENZYMATIC TREATMENT	MODIFIED PRODUCT	SOURCE OF BIAS
cap ——— OH<br>PPP ——— OH<br>P ——— OH<br>HO ——— OPO_3	1. Phosphatase<br>2. TAP treatment<br>3. RNA 5'-adapter ligation	——— OH<br>HO ——— OH<br>HO ——— OH<br>HO ——— OPO_3	1. Intramolecular ligation<br>2. RNA 5'-structural accessibility<br>3. Adapter sequence and chemistry
P ——— OH<br>cap ——— OH<br>PPP ——— OH<br>HO ——— OPO_3	1. RNA 5'-adapter ligation	——— OH<br>cap ——— OH<br>PPP ——— OH<br>HO ——— OPO_3	1. Intramolecular ligation<br>2. RNA 5'-structural accessibility<br>3. Adapter sequence and chemistry
P ——— OH<br>PPP ——— OH<br>cap ——— OH<br>HO ——— OPO_3	1. Polyphosphatase<br>2. RNA 5'-adapter ligation	——— OH<br>——— OH<br>cap ——— OH<br>HO ——— OPO_3	1. Intramolecular ligation<br>2. RNA 5'-structural accessibility<br>3. Adapter sequence and chemistry
P ——— OH<br>PPP ——— OH<br>cap ——— OH<br>HO ——— OPO_3	1. Pyrophosphohydrolase<br>2. RNA 5'-adapter ligation	——— OH<br>——— OH<br>cap ——— OH<br>HO ——— OPO_3	1. Intramolecular ligation<br>2. RNA 5'-structural accessibility<br>3. Adapter sequence and chemistry
cap ——— OH<br>PPP ——— OH<br>P ——— OH<br>HO ——— OPO_3	1. TAP treatment<br>2. RNA 5'-adapter ligation	——— OH<br>——— OH<br>——— OH<br>HO ——— OPO_3	1. Intramolecular ligation<br>2. RNA 5'-structural accessibility<br>3. Adapter sequence and chemistry

cap — OH<br>PPP — OH<br>P — OH<br><br>HO — OPO_3	1. Terminator 5’-Exonuclease<br>2. TAP treatment<br>3. RNA 5’-adapter ligation	— OH<br>— OH<br><br>5’-monophosphorylated RNAs are digested<br>HO — OPO_3	1. Intramolecular ligation<br>2. RNA 5’-structural accessibility<br>3. Adapter sequence and chemistry
P — OH<br>HO — OPO_3<br><br>cap — OH<br>PPP — OH	1. T4 PNK treatment<br>2. RNA 5’-adapter ligation	— OH<br>— OH<br><br>cap — OH<br>PPP — OH	1. Intramolecular ligation<br>2. RNA 5’-structural accessibility<br>3. Adapter sequence and chemistry
cap — OH<br>PPP — OH<br>P — OH<br><br>HO — OPO_3	1. Poly(A)polymerase/CTP	cap — $CCCCC_n$<br>PPP — $CCCCC_n$<br>P — $CCCCC_n$<br><br>HO — OPO_3	1. Posttranscriptional RNA 3’ modification<br>2. RNA structural accessibility<br>3. RNA 5’-end modification
cap — OH<br>PPP — OH<br>P — OH<br><br>HO — OPO_3	1. Poly(A) polymerase/ATP	cap — $AAAAA_n$<br>PPP — $AAAAA_n$<br>P — $AAAAA_n$<br><br>HO — OPO_3	1. Posttranscriptional RNA 3’ modification<br>2. RNA structural accessibility<br>3. RNA 5’-end modification

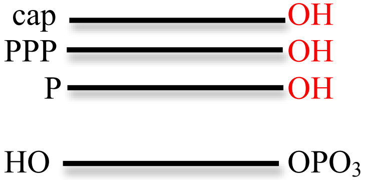	1. RNA 3’-adapter ligation	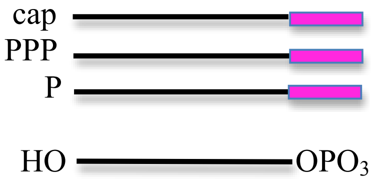	1. Posttranscriptional RNA 3’-modification<br>2. RNA structural accessibility<br>3. RNA 5’-end modification<br>4. Intramolecular ligation

STARTING MATERIAL: The starting pool of cellular RNAs with different 5’- and 3’-end modifications is schematically indicated. Abbreviations for the various modifications: OH: hydroxyl, OPO_3: 2’-3’-cyclic phosphate, ppp: triphosphate, p: monophosphate, and cap: 5' cap structure. 5’- and 3’-end modifications shown in red are substrates for enzymatic treatment in the listed reactions. ENZYMATIC TREATMENT: Different enzymatic pre-treatments prior to RNA 5’-ligation to enrich for different RNA subtypes. The last three rows indicate RNA classes accessible for 3’-end tailing [oligo(A) or oligo(C) tailing] and adapter ligation. Notably, to modify the 3’-end of 2’-3’-cyclic phosphate (OPO_3)-containing RNA molecules, T4 PNK treatment has to be performed prior to 3’-tailing or adapter ligation. MODIFIED PRODUCT: RNA classes accessible for adapter ligation after the respective 5’-end pre-treatments are schematically represented. Blue lines indicate the ligated RNA 5’-adapters. Pink represents RNA 3’-tailing or 3'-adapter ligation. SOURCE OF BIAS: List of potential distorting influences.
