# Supplementary material for: Biases in small RNA deep sequencing data
Source: Nucleic Acids Res. 2013 Nov 5;42(3):1414–26. doi: 10.1093/nar/gkt1021 (PMC3919602; doi:10.1093/nar/gkt1021)
Supplement: Supplementary Data [file supp_gkt1021_nar-01932-survey-d-2013-File004.doc]

**Supplementary Material**

**Summary**

Given the multitude of potential influences that act to distort RNA deep sequencing data and the extreme variability encoded within RNA primary and secondary structure it is very difficult to avoid bias completely (see also *Discussion* in the main text) . From what we know in chemistry, reactions depending on specific substrate combinations proceed with different efficacies. Variation in the relative efficacy of reacting components during end-modification dictates the amount and frequency of RNA molecules sequenced. As a result, RNA expression levels calculated in deep sequencing experiments might not always mirror absolute values but also reflect method-inherent differences in the efficiencies of cDNA synthesis (see also *Discussion* within in the main text). The choice of potential strategies to overcome or at least minimize bias has to consider the goal of the study. For example, to avoid exclusion of certain RNA species in RNA discovery, several methods of end modification and cDNA synthesis should be combined (see also *Discussion* in the main text) . Whenever comparative inter-sample analysis is intended to calculate relative changes of RNAs between samples, strictly identical chemistries and methods might help to exclude or at least greatly minimize bias between data sets . Therefore, barcoding might be critical, particularly when introduced by ligation . However, substrate combinations of reacting RNA termini and adapter molecules to modify ends are considered to behave identically if monitored under the same conditions. To date, the inter-sample quantification of expression levels might not enable the monitoring of all RNA species, but when performed under strictly comparative conditions does permit appropriate quantification of RNAs amenable to sequencing (see also *Discussion* in the main text) . In conclusion, inter-sample alterations in RNA expression might actually be more significant than thought, because the distorting influence due to specific chemistries and methods might be negligible if monitored under identical conditions in both samples (see also *Discussion* in the main text). On the other hand, the analysis of entire transcriptomes for discovery of all cellular RNAs is hampered by the extremes of different transcripts, and consequently has to accommodate variable RNA-specific requirements to generate cDNA. Similar technical difficulties are associated with the relative ranking of RNA intra-sample expression levels (see also *Discussion* in the main text).

Once more, analysis of entire transcriptomes to cover complete sets of cellular RNAs calls for the parallel application of different methods and chemistries to increase the probability of productive end modification. Of course, the parallel application of different methods is considered to widen the pool of accessible RNAs that are subjected to productive cDNA synthesis. An increased diversity of RNA species converted to cDNA due to the combination of different methods and reagents raises the corresponding library complexity (see also *Discussion* in the main text). Therefore, tailing-based approaches and adapter ligation, in particular when 2’-O-methylated RNA 3’-termini are also subjected to deep sequencing, might have to be employed in combination. Furthermore, not only various techniques, but also variation in the reagents helps to increase cloning efficacy. For instance, pools of different adapter oligonucleotide sequences have proven beneficial to minimizing bias . Because the complete identification of all RNAs is difficult to achieve, methods to enrich for RNA sub-sets of interest might prove beneficial. Therefore, specific chemical pre-treatment to enable enrichment according to experimental design is subject to further discussion below. Enrichment further enables increased target-specific sequencing depth.

Supplementary Table S1 summarizes specific cloning procedures used frequently to enrich sRNA-seq libraries with different subsets of RNA species.

*RNA secondary structures*

## A major source of RNA deep sequencing bias results from influences of RNA secondary structure, thus distortioning the representation of RNAs within data sets (see also *RNA secondary structure influences RNA 3’-end tailing* and *Impact of RNA secondary structure and adapter/RNA co-folding on ligation* within the main text) .

To minimize bias due to structure-derived influence, sample denaturation is of prime importance. It might prove beneficial to conduct RNA denaturation in thin-walled reaction tubes to ensure fast heat dispersal, and is of particular importance for quick chilling after heat denaturation. Again, relative intra-sample expression level ranking is likely associated with uncertainty, but sample comparability may be ensured by reducing methodological variability.

Tobacco Acid Pyrophosphatase *(TAP) pre-treatment*

TAP pre-treatment enables access not only to triphosphorylated transcripts but also to capped RNAs (Supplementary Table S1, see also *Specific biochemistry of RNA 5’-termini and enrichment of various RNA classes* in the main text) . Often by-products of RNA processing and endonuclease-mediated cleavage escape identification, as they are 5’-hydroxylated transcripts and are chemically inert to direct RNA 5’-modification. Therefore, experimental exclusion of processing side-products increases sequencing depth of target RNAs. Recent data uncovered the potential influence of RNA 5’-end phosphorylation states on the efficacy of RNA 3’-end modification. (Supplementary Table S1, see also *Specific biochemistry of RNA 5’-termini and enrichment of various RNA classes* within the main text) . The enzymatic pre-treatment helps to reduce influences derived from RNA 5’-biochemistries and should be conducted prior to 3’-end modification (4). In conclusion, TAP treatment is a necessary pre-condition for identifying the maximum number of different RNA species. (Supplementary Table S1, see also *Specific biochemistry of RNA 5’-termini and enrichment of various RNA classes* within the main text).

*Enrichment for capped or triphosphorylated RNAs.*

A minor modification of the above method permits specific enrichment of capped or triphosphorylated transcripts *.* Sample pre-treatment with terminator 5’-exonuclease to specifically digest 5’-monophosphorylated RNAs, and subsequent application of TAP permits selective access to capped or triphosphorylated RNAs (Supplementary. Table S1, see also *Specific biochemistry of RNA 5’-termini and enrichment of various RNA classes* within the main text).

*Inter-sample comparison to analyze RNA 5’-phosphorylation states*

An interesting approach to map start sites of transcription in prokaryotes relies on the bio-computational analysis of sample read compositions in response to different enzymatic treatments (Supplementary Table S1, see also *Specific biochemistry of RNA 5’-termini and enrichment of various RNA classes* within the main text) *.* Because samples subjected to direct end-modification without enzymatic pre-treatment enable the identification of monophosphorylated RNA 5’-termini, and as terminator 5’-exonuclease application in combination with subsequent TAP treatment enriches for triphosphorylated RNAs only, bio-computational analysis enables the identification of actual phosphorylation states. The frequency of specific RNA 5’-termini in response to both treatments is calculated and compared after normalization . If read frequencies are higher in terminator 5’-exonuclease-treated samples, 5’-termini are considered to be triphosphorylated .

*Direct ligation*

In cases where direct examination of 5’-mono-phosphorylated RNA termini is intended, direct RNA 5’-end ligation provides specific enrichment for 5’-monophosphorylated RNAs. Because all other biochemistries at RNA 5’-termini require enzymatic pre-treatment prior to end modification, direct cloning permits specific enrichment of 5’-monophosphorylated RNAs (Supplementary Table S1).

*Enrichment of tri- or monophosphorylated RNAs*

Polyphosphatase or Pyrophosphohydrolase pre-treatment is reported to enable specific enrichment of triphosphorylated or monophosphorylated RNAs alike (Supplementary Table S1, see also *Specific biochemistry of RNA 5’-termini and enrichment of various RNA classes* within the main text) . However, neither enzyme triggers cleavage of pyrophophate bonds in capped RNAs .

*Polynucleotide kinase treatment to identify 5’ hydroxylated RNAs.*

5’-hydroxylated RNA termini are enriched by PNK pre-treatment. The resulting monophosphorylation of the originally 5’-hydroxylated RNAs permits RNA 5’-end ligation and excludes capped or triphosphorylated RNA termini. Specific inter-sample comparisons to identify enrichment between samples subjected to direct cloning and samples that are modified by PNK treatment enables the identification of hydroxylated RNAs.

*RNA 3’ end tailing and RNA 3’ ligation*

## Recent analysis based on deep sequencing as read out uncovered no primary nucleotide preference of poly(A) polymerase . However, a detailed biochemical investigation to delineate the time-course of RNA tailing reported altered reaction kinetics as a consequence of the 3’-terminal nucleotide (see also *RNA 3’-tailing* within the main text) . In agreement with most biochemical data, 1-2 hours of RNA tailing sufficed for efficient end modification of most test RNAs (see also *RNA 3’-tailing* within the main text) . This time is an approximation, as the biochemical analysis relied on only a few test RNAs . Increased incubation times help to modify difficult templates and increase the library complexity (see also *RNA 3’-tailing* within the main text). A further advantage of tailing-based approaches is the exclusion of adapter dimers that severely influence library complexity and might be dominating if RNA starting material is limited (see also *Adapter-adapter ligation disturbs sRNA-seq* with the main text) . In addition, tailing-based methods do not require gel purification away from adapter leftovers to decrease dimer formation. The exclusion might not only simplify protocols but is advantageous when only limited amounts of RNA starting material are available (see also *RNA 3’-tailing and Adapter-adapter ligation disturbs sRNA-seq* with the main text).

*A-versus C-tailing to enable full-length cDNA generation*

Apart from the chemical nature of the RNA termini subject to the actual modification, the experimental design itself further impacts the choice of RNA modification method. RNAs generated by retro-transposition occasionally harbor gene-internal, A-rich sequence tracts . Unintended gene internal oligo(T) priming in reverse transcription would thereby prohibit the application of RNA 3’-A-tailing, if identification of native RNA termini is desired . RNA C-tailing is a viable alternative and was also the method of choice to map 3’-termini of processed mitochondrial rRNA of Plasmodium falciparum . In conclusion, RNA 3’ tailing is generally considered to deliver robust and less biased results and might be preferred when the experimental subject permits (see also *RNA 3’-tailing* within the main text).

*Adapter ligation when 2’ O-methylated templates are included in deep sequencing surveys.*

## A major drawback of poly(A) polymerase-mediated approaches relates to the inefficient end modification of 2’-O-methylated RNA 3’-termini. However, 2’-O-methylated test RNAs are captured effectively by RNA 3’-ligation, in particular, when the reactions are supplemented by polyethylenglycol (PEG) (see also *RNA 3’-tailing* and *RNA ligation* within the main text).

*Adapter ligation*

RNA ligation displays primary sequence- and RNA secondary structure-related distortions. Irrespective of the *de facto* source and when RNA 3’-adapter ligation is not avoidable, increased adapter sequence variability to increase the likelihood of productive RNA ligation has been suggested to minimize bias (see also *RNA ligation* and *Discussion* within the main text) . Furthermore, in addition to changes in primary sequence, the application of different adapter chemistries, such as DNA, RNA, and chimeric oligonucleotides, is recommended (see also *RNA ligation* and *Discussion* within the main text) . Various strategies that employ adapter combinations were reported recently (see also *Discussion* and *RNA ligation* within the main text) . In addition, kits to enable the in-house production of various 5’-adenylated adapter oligonucleotides permitted the cost-effective application of many adapters in parallel .

It is also recommended that adapter oligonucleotides utilized in RNA 3’-end ligations carry modified 3’-termini devoid of 3’-terminal hydroxyl functions. For custom designed synthesis, companies therefore offer ddC-end modification to effectively block self-ligation. The use of specific reagents, in particular RNA ligase 2 and derivatives thereof, to reduce side reactions is elaborated in detail (see also *RNA ligation* within the main text). Strategies to avoid unintended adapter-adapter ligation are described within the main text.

References

1. Hafner, M., Renwick, N., Brown, M., Mihailovic, A., Holoch, D., Lin, C., Pena, J.T., Nusbaum, J.D., Morozov, P., Ludwig, J. *et al.* (2011) RNA-ligase-dependent biases in miRNA representation in deep-sequenced small RNA cDNA libraries. *Rna*, **17**, 1697-1712.

2. Sorefan, K., Pais, H., Hall, A.E., Kozomara, A., Griffiths-Jones, S., Moulton, V. and Dalmay, T. (2012) Reducing ligation bias of small RNAs in libraries for next generation sequencing. *Silence*, **3**, 4.

3. Zhuang, F., Fuchs, R.T., Sun, Z., Zheng, Y. and Robb, G.B. (2012) Structural bias in T4 RNA ligase-mediated 3'-adapter ligation. *Nucleic Acids Res*, **40**, e54.

4. Raabe, C.A., Hoe, C.H., Randau, G., Brosius, J., Tang, T.H. and Rozhdestvensky, T.S. (2011) The rocks and shallows of deep RNA sequencing: Examples in the Vibrio cholerae RNome. *Rna*, **17**, 1357-1366.

5. Alon, S., Vigneault, F., Eminaga, S., Christodoulou, D.C., Seidman, J.G., Church, G.M. and Eisenberg, E. (2011) Barcoding bias in high-throughput multiplex sequencing of miRNA. *Genome Res*, **21**, 1506-1511.

6. Eminaga, S., Christodoulou, D.C., Vigneault, F., Church, G.M. and Seidman, J.G. (2013) Quantification of microRNA expression with next-generation sequencing. *Curr Protoc Mol Biol*, **Chapter 4**, Unit 4 17.

7. Jayaprakash, A.D., Jabado, O., Brown, B.D. and Sachidanandam, R. (2011) Identification and remediation of biases in the activity of RNA ligases in small-RNA deep sequencing. *Nucleic Acids Res*, **39**, e141.

8. Suzuki, Y., Yoshitomo-Nakagawa, K., Maruyama, K., Suyama, A. and Sugano, S. (1997) Construction and characterization of a full length-enriched and a 5'-end-enriched cDNA library. *Gene*, **200**, 149-156.

9. Fromont-Racine, M., Bertrand, E., Pictet, R. and Grange, T. (1993) A highly sensitive method for mapping the 5' termini of mRNAs. *Nucleic Acids Res*, **21**, 1683-1684.

10. Mandl, C.W., Kunz, C. and Heinz, F.X. (1991) Presence of poly(A) in a flavivirus: significant differences between the 3' noncoding regions of the genomic RNAs of tick-borne encephalitis virus strains. *J Virol*, **65**, 4070-4077.

11. Sharma, C.M., Hoffmann, S., Darfeuille, F., Reignier, J., Findeiss, S., Sittka, A., Chabas, S., Reiche, K., Hackermuller, J., Reinhardt, R. *et al.* (2010) The primary transcriptome of the major human pathogen Helicobacter pylori. *Nature*, **464**, 250-255.

12. Albrecht, M., Sharma, C.M., Dittrich, M.T., Muller, T., Reinhardt, R., Vogel, J. and Rudel, T. (2011) The transcriptional landscape of Chlamydia pneumoniae. *Genome Biol*, **12**, R98.

13. Bessman, M.J., Walsh, J.D., Dunn, C.A., Swaminathan, J., Weldon, J.E. and Shen, J. (2001) The gene ygdP, associated with the invasiveness of Escherichia coli K1, designates a Nudix hydrolase, Orf176, active on adenosine (5')-pentaphospho-(5')-adenosine (Ap5A). *J Biol Chem*, **276**, 37834-37838.

14. Deana, A., Celesnik, H. and Belasco, J.G. (2008) The bacterial enzyme RppH triggers messenger RNA degradation by 5' pyrophosphate removal. *Nature*, **451**, 355-358.

15. Fujimura, T. and Esteban, R. (2012) Cap snatching of yeast L-A double-stranded RNA virus can operate in trans and requires viral polymerase actively engaging in transcription. *J Biol Chem*, **287**, 12797-12804.

16. Munafo, D.B. and Robb, G.B. (2010) Optimization of enzymatic reaction conditions for generating representative pools of cDNA from small RNA. *Rna*, **16**, 2537-2552.

17. Kawano, M., Kawazu, C., Lizio, M., Kawaji, H., Carninci, P., Suzuki, H. and Hayashizaki, Y. (2010) Reduction of non-insert sequence reads by dimer eliminator LNA oligonucleotide for small RNA deep sequencing. *Biotechniques*, **49**, 751-755.

18. Vigneault, F., Ter-Ovanesyan, D., Alon, S., Eminaga, S., D, C.C., Seidman, J.G., Eisenberg, E. and G, M.C. (2012) High-throughput multiplex sequencing of miRNA. *Curr Protoc Hum Genet*, **Chapter 11**, Unit 11 12 11-10.

19. Brosius, J. (1999) RNAs from all categories generate retrosequences that may be exapted as novel genes or regulatory elements. *Gene*, **238**, 115-134.

20. DeChiara, T.M. and Brosius, J. (1987) Neural BC1 RNA: cDNA clones reveal nonrepetitive sequence content. *Proc Natl Acad Sci U S A*, **84**, 2624-2628.

21. Raabe, C.A., Sanchez, C.P., Randau, G., Robeck, T., Skryabin, B.V., Chinni, S.V., Kube, M., Reinhardt, R., Ng, G.H., Manickam, R. *et al.* (2010) A global view of the nonprotein-coding transcriptome in Plasmodium falciparum. *Nucleic Acids Res*, **38**, 608-617.

22. Zhelkovsky, A.M. and McReynolds, L.A. (2011) Simple and efficient synthesis of 5' pre-adenylated DNA using thermostable RNA ligase. *Nucleic Acids Res*, **39**, e117.
